# Book Reviews

**Published:** 1903-08

**Authors:** 


### BOOK REVIEWS.

American Edition of Nothnagel's Practice. I. Diseases of the stomach. by Dr. F. Riegel, of Giessen. Edited, with additions, by Charles G. Stockton, M. D., Professor of Medicine in the University of Buffalo. Octavo, 835 pages, illustrated, including six full-page plates, Philadelphia, New York, London: W. B. Saunders & Company. 1903. (Cloth, $5.00 net; half morocco, $6.00 net.) II. Diseases of the Pancreas, Suprarenal Capsules, and Liver. By Drs. L. Oser and E. Neusser, of Vienna; and Drs. H. Quincke and G. Hoppe-Seyler, of Kiel. Edited, with additions, by Frederick A. Packard, M. D., late Physician to the Pennsylvania and to the Children's Hospitals, Philadelphia; and Reginald H. Fitz, M. D.; Hersey Professor of the Theory and Practice of Physic, Harvard University Medical School, Boston. Octavo, 918 pages, illustrated. Philadelphia, New York, London: W. B. Saunders & Company. 1903. (Cloth, $5.00 net; half morocco, $6.00 net.)

I. This volume is, without question, the most comprehensive work relating to diseases of the stomach that has been published though, it must be confessed, Hemmeter gives it a neck-and-neck race. It is impossible to examine the literature pertaining to these ailments without encountering the names of Ewald, Boas, Leube, Einhorn, Hemmeter, Stockton, and now Riegel, Jones, and a few others, are to be reckoned with as lately having contributed to the present improved understanding of gastric maladies.

The employment of the stomach-tube for diagnostic purposes marked a distinct change in stomachal therapy, and to Leube belongs the credit of having introduced this method of diagnosis. Lavage of the stomach soon followed, as a natural consequence, and has become an accepted method in certain conditions. Nevertheless, it must be skilfully performed else it may fail to benefit or, indeed, may do harm. Riegel gives appropriate directions covering all the important points pertaining to lavage. The surgical treatment of these affections is considered at some length, mainly to point out which are amenable to the knife and when to invoke its aid, for it must be admitted that the specialist must make the diagnosis and determine when the surgeon's aid is needful. So far the principal conditions that offer the most opportunity for surgical intervention are cancer of the pylorus and perforated ulcer. The work of Hewitt, of Guelph, Ont., has been considerable in the latter condition, and we look forward to the time in the near future when it will be recognised as a contribution of valuable experience to the subject.

One of the main conditions that today claims the attention of the physician is hyperclorhydria. It is afflicting those in the higher walks of life to an astonishing degree, and is not easily controlled or cured because so much of success depends upon careful regulation of the diet,—a discipline that most persons afflicted rebel against. This author is very clear upon this point as, indeed, he is upon the entire subject of hyperacidity. The volume is full of the latest and best information, and it is fortunate that so competent an editor as Professor Stockton has been obtained. His additions and annotations most thoroughly Americanise the work and give expression to the last thought on all important points.

II. Diseases of the liver, pancreas, and suprarenal capsules, which form the topics considered in the second volume of Nothnagel's practice now under examination, have become invested with increased importance of late, especially the maladies of the latter two. The liver is an old offender, yet for ages it has been charged with misdeeds that it was innocent of committing. Cholelithiasis, however, is one of the offenses that the liver has been guilty of from a very early day, yet not until recently has its significance become apparent. It is quite true that the older writers recognised gallstone disease, but it is also quite true that until Sims and Kocher introduced its operative treatment, no advance had been made in its therapy, since the days of sweet-oil and mineral waters as solvents.

Quincke and Hoppe-Seyler have set forth in the volume the most complete history of diseases of the liver we have seen, and all the modern methods of diagnosis and treatment are detailed with intelligent accuracy. Very properly, however, they refer to Kehr, Languenbuch, and Riedel for operative technique in the surgery of the liver and gall-bladder.

Professor Oser has presented a monograph on diseases of the pancreas, the value of which cannot be overestimated. If surgery may be credited with having influenced the advance in knowledge concerning diseases of the liver, so, too, must it receive like credit for improvement in diseases of the pancreas. Nearly all the best information on this subject has sprung up during the last ten years, and surgeons, both American and foreign, have been large contributors to it.

Diseases of the suprarenal capsules are dealt with by Professor Neusser in the most scholarly manner. Addison in 1855, described a disease of these bodies, which has become known by his name the world over, which, too, comprises about all that was known of suprarenal disease, until lately. In this monograph the entire subject receives adequate attention.

These two volumes serve to further accentuate the fact that Nothnagel's practice must become a standard guide for the clinician for some time to come.

---

Twentieth Century Practice. An International Encyclopedia of Modern Medical Science by Leading Authorities of Europe and America. Edited by Thomas L. Stedman, M. D., New York City. In 21 volumes. Volume XXI.—Supplement. New York: William Wood & Co. 1903. (Price, cloth, $5.00.)

The twentieth century is beginning with an energy no less strenuous in medicine than in other fields of science or business. Since the completion of the series of volumes, known as the "Twentieth Century Practice," many advances have taken place, requiring the publication of a supplementary volume to keep the work abreast of the immediate present. If we recall the improvements in radiotherapy, the discovery of the agency of the mosquito in the transmission of certain infectious diseases, advanced studies in hematology, bacteriology, and the investigations relating to cancer, together with many other researches that have been made, or are making, bearing upon important diseases, one can quite appreciate the presentation of this volume, in which all the recent work in these directions is recorded.

The section on yellow fever by General Sternberg, will prove of special interest. No man is more competent than he to deal with the subject, and it is a credit to the medical department of the Army, over which General Sternberg presided so long and with such distinction, that through its agency and by one of its own distinguished members, Cuba was rid of an infectious disease that had existed without interruption for three centuries. The name of Walter Reed is inseparably connected with the eradication of yellow fever in Cuba, and General Sternberg tells the whole story in his brochure herein published. It appears that the general, himself, first suggested to Dr. Reed that special attention should be given to the possibility of the transmission of the disease by some insect, having previously studied the subject from this viewpoint in tropical countries.

The volume is replete with material of essential interest, and will be sought eagerly by those who possess the first twenty volumes, while every one who enjoys scientific research, may read it with genuine satisfaction.

---

International Clinics. A Quarterly of Illustrated Clinical Lectures and especially prepared original Articles on Treatment, Medicine, Surgery, Neurology, Pediatrics, Obstetrics, Gynecology, Orthopedics, Pathology, Dermatology, Ophthalmology, Otology, Rhinology, Laryngology, Hygiene, and other topics of interest to Students and Practitioners, by leading members of the medical profession throughout the world. Edited by A. O. J. Kelly, A.M., M. D., Philadelphia, with the collaboration of Wm. Osler, M. D., Baltimore; John H. Musser, M. D., Philadelphia; Jas. Stewart, M. D., Montreal; John B. Murphy, M. D., Chicago; Thomas M. Rotch, M. D., Boston; John G. Clark, M. D., Philadelphia; James J. Walsh, M. D., New York; J. W. Ballantyne, M. D., Edinburgh; John Harold, M. D., London; Edmund Landolt, M. D., Paris, and Richard Kretz, M. D., Vienna, with regular correspondents in Montreal, London, Paris, Berlin, Vienna, Leipsic, Brussels and Carlsbad. Volume I. Thirteenth series. 1903. Philadelphia; J. B. Lippincott Company. 1903. (Cloth, $2.00.)

The new editor of this periodical has made an admirable beginning, this being the first volume of the "Clinics," under the editorial supervision of Dr. A. O. J. Kelly. The standard set by Dr. Cattell was a high one, yet the first volume of the thirteenth series gives promise of increasing excellence. The topics considered in this number are: I. Treatment with Osler, Wilcox, Satterthwaite, Fenger, and Fussell, as contributors. 2. Medicine, with Billings and Bangs, as the authors. 3. Surgery, with Keen, Senn, Jonnesco, Ross (George G.), and Manley as lecturers. 4. Pediatrics with Thomson as sole contributor. 5. Orthopedics, to which only Shands contributes. Two special articles follow, one on functional reversion and its import in medical practice, by A. F. A. King, and the other on the general principles of embryology, by J. W. Ballantyne. Finally, comes a review of the progress of medicine, during the year 1902, prepared jointly by Edward Willard Watson, and Henry W. Cattell.

From this it will be observed that the contributors are men of fame, while the subjects appeal to the general reader. The illustrations in this volume are greater in number and better in quality than has been the case in many of the previous books.

---

Report of the Commissioner of Education for the Year 1900-1901. Volume II. Washington: Government Printing Office. 1902.

The questions that will attract special attention are the coeducation of the sexes and the present educational movement in the Philippines Islands. Each are dealt with in considerable detail, about one hundred pages apiece being allotted. Prominent educators throughout the country have been invited to express their views on the subject of coeducation, and these opinions have been printed, in whole or in part, in this volume. There are also some notes on coeducation in foreign countries. In view of the general importance of the problem, it would be well for all interested in it, either practically or theoretically, to read the chapter herein devoted to its consideration.

As to the progress of education in the Philippines, much light is thrown upon the subject in the chapter printed in this book relating to it. The military government necessarily was compelled to deal with educational questions at first, and now the civil government, with Judge Taft, at its head, is grappling with this problem. The difficulties are herein set forth, but the work seems to be progressing in a satisfactory manner. The remaining chapters of the book consider routine questions, relating to professional, normal, secondary, and private schools, as well as some other topics of interest to educators.

---

A System of Physiologic Therapeutics. A Practical Exposition of the Methods, other than Drug-giving, useful for the Prevention of Disease and in the Treatment of the Sick. Edited by Solomon Solis Cohen, A.M., M. D., Senior Assistant Professor of Clinical Medicine in Jefferson Medical College. Volume X. Pneumotherapy, including Aërotherapy and inhalation Methods and Therapy, by Paul Louis Tissier, Chief of Clinic in the Faculty of Medicine of the University of Paris. Octavo, pp. 494. Illustrated. Philadelphia: P. Blakiston's Son & Co. 1903. (Price for the set, $27.50 net.)

The terms "pneumotherapy" and "aerotherapy," as herein applied, are concisely defined by the author. He says: The term pneumotherapy is applied to the use of respired gases for therapeutic purposes, directly or as carriers of medicinal agents; aerotherapy is a branch of pneumotherapy, dealing with atmospheric air.

These methods of treating the breathing organs have been too long neglected, or but partly applied, and that, perhaps, indifferently. A careful study of the treatise will serve to stimulate renewed energy in this direction, no doubt, and particularly to develop scientific application of medicinal gases and pure air to the treatment of the diseases to which they may be applied with appropriateness. It is hoped, too, that a better understanding of these subjects will serve to lift this treatment out of the hands of the quacks, "Koch lung cures," and all such swindling concerns. A careful study of this treatise is strongly advised. It is the most complete presentation of the subject that has appeared as yet.

Progressive Medicine. Fifth Annual Series. Volume II., June, 1903. A Quarterly Digest of Advances. Discoveries and Improvements in the Medical and Surgical Sciences. Edited by Hobart Amory Hare, M. D., Professor of Therapeutics and Materia Medica in the Jefferson Medical College of Philadelphia. Octavo, 427 pages, with 46 illustrations. Lea Brothers & Co., Philadelphia and New York. (Per volume, $2.50, by express prepaid. Per annum, in four cloth-bound volumes, $10.00.)

The first section of this volume considers surgery of the abdomen, including hernia, and is prepared by William B. Coley. It contains a description of the newer methods relating to the surgery of hernia, the stomach, the intestines, the liver and biliary passages, the kidney, and some other organs or tissues. The illustrations are plentiful and delineative of the text,—the whole section being in keeping with the representative surgeon, who prepared it. The next section, gynecology, is edited by John G. Clark. There have been no remarkable discoveries of late in this department of medicine, yet considerable good work has been done in the line of perfecting methods or correcting errors. Cancer of the uterus, the urinary system in women, retrodisplacements, and inflammatory affections of the pelvic viscera, are the chief subjects presented here. The next section offers discourse on diseases of the blood and ductless glands, the hemorrhagic diseases, and diseases of metabolism, and is arranged under the supervision of Alfred Stengel. Finally, ophthalmology constitutes a division under the marshalship of Edward Jackson. The best of the late literature is herein epitomised and handed out to a busy profession by some of the masters, not only in their several special lines of work, but by literary experts, who glean only the wheat, letting the chaff blow away.

The Care of the Baby. A Manual for Mothers and Nurses, containing Practical Directions for the Management of Infancy and Childhood in Health and in Disease. By J. P. Crozer Griffith, M. D., Clinical Professor of Diseases of Children in the Hospital of the University of Pennsylvania; Physician to the Children's Hospital, Philadelphia. Third edition, thoroughly revised. Duodecimo volume of 436 pages, fully illustrated. Philadelphia, New York, London: W. B. Saunders & Co. 1903. (Cloth, $1.50 net.)

This little treatise is without doubt the best of its kind in existence. Physicians acquainted with it are constantly recommending it to young mothers as a safe guide in the rearing of children. The third edition has been carefully revised and various additions have been made. It is a pleasure to know that the book is meeting a deserved success.

Uterine and Tubal Gestation. A Study of the Embedding and Development of the Human Ovum, the early Growth of the Embryo, and the Development of the Synctium and Placental Gland. By Samuel Wyllis Bandler, M. D., Instructor in Gynecology in New York Post-Graduate Medical School. Small octavo, pp. 170. Illustrated by 93 drawings. New York: William Wood & Company. 1903. (Price, $1.50.)

Since Lawson Tait wrote so graphically upon the surgery of ectopic pregnancy it has had a new meaning to the professional world, and it has been studied with new interest by physicians everywhere. This brochure takes up the study from a different viewpoint than that usually adopted. It begins by considering the essentials of uterine gestation, then takes up tubal gestation, and finally discourses upon ovarian and placental secretion. These are correlated parts of practically one and the same subject; hence, may be studied in consecutive order.

This author presents an interesting essay on the relation of ovulation to menstruation and discourses with much intelligent force upon the processes antedating uterine gestation. The development of the human ovum also is treated with clearness and much interesting discussion is had with regard to the embedding of the ovum. Many of the views herein set forth will become subjects for further investigation and remain to be established or to be set aside; nevertheless, they deserve careful examination by every obstetrician as well as every gynecologist. It will be a long time before the last word is said on uterine and tubal gestation, yet this author has contributed not a little toward solving the mysteries that surround these delicate processes.

---

Medical Jurisprudence, Insanity, and Toxicology. By Henry C. Chapman, M. D., Professor of Institutes of Medicine and Medical Jurisprudence in the Jefferson Medical College, Philadelphia. Third edition, thoroughly revised, greatly enlarged, and entirely reset. Duodecimo volume of 329 pages, fully illustrated, including four colored plates. Philadelphia, New York, London: W. B. Saunders & Company. 1903. (Cloth, $1.75 net.)

It is not surprising that such an excellent manual should require reprinting and revising at comparatively short intervals. It deals with certain questions that too often are neglected by physicians, because they are not met with frequently perhaps; nevertheless, when confronted they often prove perplexing in the extreme. Therefore, it becomes necessary to be prepared for the emergency, and this book will serve the purpose of supplying the needful preparation. The author has had just the personal experience in his six years' service as coroner physician in a large city, that enables him to present the practical side of medical jurisprudence.

The size of the book is in its favor, too, because it is easy to carry along with one when going to court as a witness, and to consult while waiting to be called to the witness-box. This edition is somewhat larger than its predecessors, has been rearranged, revised, and in other respects made to conform to the present needs. It is, in all respects, therefore, the most complete and best planned manual on medico-legal questions that is before the profession at the present time.

---

Diseases of the Heart and Arterial System. Designed to be a Practical Presentation of the Subject for the Use of Students and Practitioners of Medicine. By Robert H. Babcock, A.M., M. D., Professor of Clinical Medicine and Diseases of the Chest, College of Physicians and Surgeons, Medical Department of the Illinois State University, Chicago. Octavo, pp. 874. With three colored plates and 139 illustrations. New York and London: D. Appleton & Company. 1903. (Price, $6.00.)

This book is as large as such a treatise on the theory and practice of medicine thirty or forty years ago; and yet it is devoted to the consideration of a single organ of the body, albeit the most important of the viscera.

The studies of the diseases of the heart have been regarded as among the most difficult in the entire category of human maladies, but modern methods and recent investigations have served to simplify, in a measure, the perplexities surrounding this important segment of medicine. Such books as this play a significant part in developing a simpler and clearer method of study. The distinguishing features of this treatise are its practical character, the omission from its pages in large part of mere theories, a clear but limited use of anatomy and physiology, ample consideration of physical signs in the diagnosis of heart affections, simple phraseology, familiar terminology, and special consideration of treatment in an amplitude of detail not heretofore observed in works on the heart.

If we should undertake to present with analytical detail the contents of this treatise in consecutive order, it would weary the reader, we fear; but we must confess, the temptation to enter into a general discussion of the subjects dealt with by the author is very great. He has grouped in a single volume all the essential facts connected with the heart and its diseases, making a study of this subject one of agreeable occupation, instead of laborious application, as heretofore has been the case, with a literature scattered throughout textbooks and magazines.

It is the best illustrated treatise on the heart and bloodvessels we have seen from the viewpoints of both quantity and quality, and in general it may be regarded with entire justice, as one of the most agreeable, if not useful, medical books of the season.

---

Practical Points in Nursing. For Nurses in Private Practice. With an Appendix containing Rules for Feeding the Sick; Receipts for Invalid Food and Beverages; Weights and Measures; Dose List; and a full Glossary of Medical Terms and Nursing Treatment. By Emily A. M. Stoney, late Superintendent of the Training School for Nurses, Carney Hospital, South Boston, Mass. Third edition, thoroughly revised. Duodecimo of 458 pages, fully illustrated, including eight colored and half-toned plates. Philadelphia, New York, London: W. B. Saunders & Company. 1903. (Cloth, $1.75 net.)

This excellent manual proves its popularity in the early appearance of the third edition. The death of its author has made necessary its revision at other hands, but the practical character of the book is not lost thereby, the changes being only such as serve to keep it abreast of the progress of modern medicine. It has become a standard in its field and should be found in every hospital library, as well as in the hands of every nurse.

---

The Expectant Mother. A Treatise on the care of Expectant Mother during Pregnancy and Child-birth and the care of the Child from Birth to Puberty. By W. Lewis Howe, M. D. Pages VIII-63. Small 12mo. F. A. Davis Company, Philadelphia. 1903. ($.50' net, delivered.)

Whenever a physician desires to place in the hands of his women patients a book which contains in small space all that a mother really needs to know to prepare herself for confinement and intelligently to rear her child thereafter, he may give them this little volume. It does not aim to make every mother her own doctor and, therefore, is just suited as a guide to the woman of limited intelligence on the subject-matter of which it treats.

